# Was there any delay in the treatment of breast cancer patients because of the COVID-19 stringency measures?

**DOI:** 10.1093/eurpub/ckac129.274

**Published:** 2022-10-25

**Authors:** F Estupiñán-Romero, J González-Galindo, N Martínez-Lizaga, J González-García, P Derycke, S Aldridge, A Faragalli, M Raimundo Maia, I Zile, E Bernal-Delgado

**Affiliations:** 1 Institute for Health Sciences in Aragon, Zaragoza, Spain; 2 Sciensano, Brussels, Belgium; 3 Population Data Science, Swansea University Medical School, Swansea, UK; 4 Biomedical Sciences and Public Health, Marche Polytechnic University, Ancona, Italy; 5 NOVA University of Lisbon, Lisbon, Portugal; 6 Center for Disease Prevention and Control, Riga, Latvia

## Abstract

**Background:**

Healthcare systems across Europe reorganized services to provide attention to COVID-19 patients. In the event of the surge of cases, countries were forced to cancel or postpone non-urgent care. The objective of this work is to investigate whether there were time-to-treatment delays in breast cancer due to April-May 2020 restrictions, and whether the delays were permanent and different across countries.

**Methods:**

Design: Quasi-experimental pre-post study with a historical control. Population: Virtually the universe of breast cancer patients receiving elective surgery, radiotherapy, hormonal therapy or chemotherapy since January 2017 (until December 2021) in the participant regions - Belgium, Marché (IT), Riga (LV), Portugal, Wales, and Aragon (ES). The main endpoint is the change in the median time-to-treatment before and after an empirical joint-point. The study variables are detailed here https://doi.org/10.5281/zenodo.5148022. Analysis: Distributed generalized additive models using https://cran.r-project.org/package=mgcv.

**Results:**

Preliminary results show that the impact in March-April 2020 time-to-treatment evolved differently across countries. For instance, while the median time from diagnosis to surgery, as the first treatment, increased from approximately 39 days (2018-2019) to more than 45 days (2020-2021) in Wales, in the Marche region (IT) the median time decreased from 52 days in 2017-2019 to 47 days in 2020. Complete analyses for the rest of the participant countries are currently undergoing.

**Conclusions:**

We have observed differences in time to treatment in women with breast cancer across countries; however, the magnitude and direction of the effect has been uneven across countries.

